# Pulse train gating to improve signal generation for *in vivo* two-photon fluorescence microscopy

**DOI:** 10.1117/1.NPh.10.4.045006

**Published:** 2023-11-06

**Authors:** Shaun A. Engelmann, Alankrit Tomar, Aaron L. Woods, Andrew K. Dunn

**Affiliations:** University of Texas at Austin, Department of Biomedical Engineering, Austin, Texas, United States

**Keywords:** two-photon microscopy, pulse gating, pulse reduction, neurovascular imaging, ultrafast lasers

## Abstract

**Significance:**

Two-photon microscopy is used routinely for *in vivo* imaging of neural and vascular structures and functions in rodents with a high resolution. Image quality, however, often degrades in deeper portions of the cerebral cortex. Strategies to improve deep imaging are therefore needed. We introduce such a strategy using the gating of high repetition rate ultrafast pulse trains to increase the signal level.

**Aim:**

We investigate how the signal generation, signal-to-noise ratio (SNR), and signal-to-background ratio (SBR) improve with pulse gating while imaging *in vivo* mouse cerebral vasculature.

**Approach:**

An electro-optic modulator with a high-power (6 W) 80 MHz repetition rate ytterbium fiber amplifier is used to create gates of pulses at a 1 MHz repetition rate. We first measure signal generation from a Texas Red solution in a cuvette to characterize the system with no gating and at a 50%, 25%, and 12.5% duty cycle. We then compare the signal generation, SNR, and SBR when imaging Texas Red-labeled vasculature using these conditions.

**Results:**

We find up to a 6.73-fold increase in fluorescent signal from a cuvette when using a 12.5% duty cycle pulse gating excitation pattern as opposed to a constant 80 MHz pulse train at the same average power. We verify similar increases for *in vivo* imaging to that observed in cuvette testing. For deep imaging, we find that pulse gating results in a 2.95-fold increase in the SNR and a 1.37-fold increase in the SBR on average when imaging mouse cortical vasculature at depths ranging from 950 to 1050  μm.

**Conclusions:**

We demonstrate that a pulse gating strategy can either be used to limit heating when imaging superficial brain regions or used to increase signal generation in deep regions. These findings should encourage others to adopt similar pulse gating excitation schemes for imaging neural structures through two-photon microscopy.

## 1 Introduction

Two-photon microscopy is routinely used to image the *in vivo* neural structure throughout the cerebral cortex in rodents at a micrometer resolution.^1^^–^^4^ Ultrafast lasers are focused using high numerical aperture objectives and raster scanned across an imaging plane to excite fluorophores that label anatomical structures. A natural limit to imaging depth arises, however, once the background fluorescence generated away from the focus approaches the level of fluorescence produced at the imaging plane^5^ or when the attenuation of excitation and emission light due to tissue scattering reduces the image signal to a point at which it is no longer detectable. The unavoidable reduction in the signal-to-background ratio (SBR) with depth critically inhibits the ability to accurately image deep anatomical structure. The issue is further exacerbated by the inherent noise that materializes during signal detection, which makes a high signal-to-noise ratio (SNR) crucial as well.

Many strategies exist to improve deep imaging in tissue. One is to use a longer excitation wavelength that is less susceptible to scattering as it travels through tissue, leading to a higher percentage of incident photons at the excitation point, ultimately increasing signal.^6^^–^^9^ Long excitation wavelengths also enable three-photon excitation with select fluorophores, which significantly reduces the amount of background florescence generated relative to two-photon imaging schemes.^10^^,^^11^ Long wavelength and three-photon imaging both extend the theoretically possible imaging depth. A more trivial solution for improving imaging at depth is to simply use a higher excitation power. This does not increase the theoretical SBR-imposed depth limit but increases the SBR in all regions above this point, ultimately facilitating imaging to the theoretical limit. However, this approach is ultimately limited by the need to limit average power levels to avoid excessive tissue heating from light absorption.^12^^,^^13^

Because heating is a function of average power and signal is related to the square of peak power (or cube in three-photon excitation), it can be useful to reduce the pulse repetition rate and use higher pulse energies while remaining below thermal damage thresholds. For this reason, low repetition rate (∼1  MHz) sources are often used for multiphoton imaging.^14^^,^^15^ These sources are commercially available but are complex and very expensive. An alternative approach is to reduce the pulse delivery from high repetition rate, high average power ultrafast lasers through pulse picking or pulse gating. There has not yet been a thorough quantification of the improvements brought forth by pulse reduction while holding the pulse characteristics (pulse width and pulse spectrum) constant for *in vivo* imaging. Our aim with this work is to provide such a comparison using a high power, relatively inexpensive, high repetition rate (80 MHz) excitation source and a pulse gating system. Past work with similar strategies has either been limited to *ex vivo* imaging^16^^,^^17^ or performed with an excitation system in which pulse characteristics varied with the repetition rate, complicating the interpretation of the results.^18^ What is presented here should encourage the adoption of similar pulse train gating schemes for *in vivo* deep brain imaging.

## 2 Methods

### 2.1 Ultrafast Pulse Gating System

**Fig. 1:**
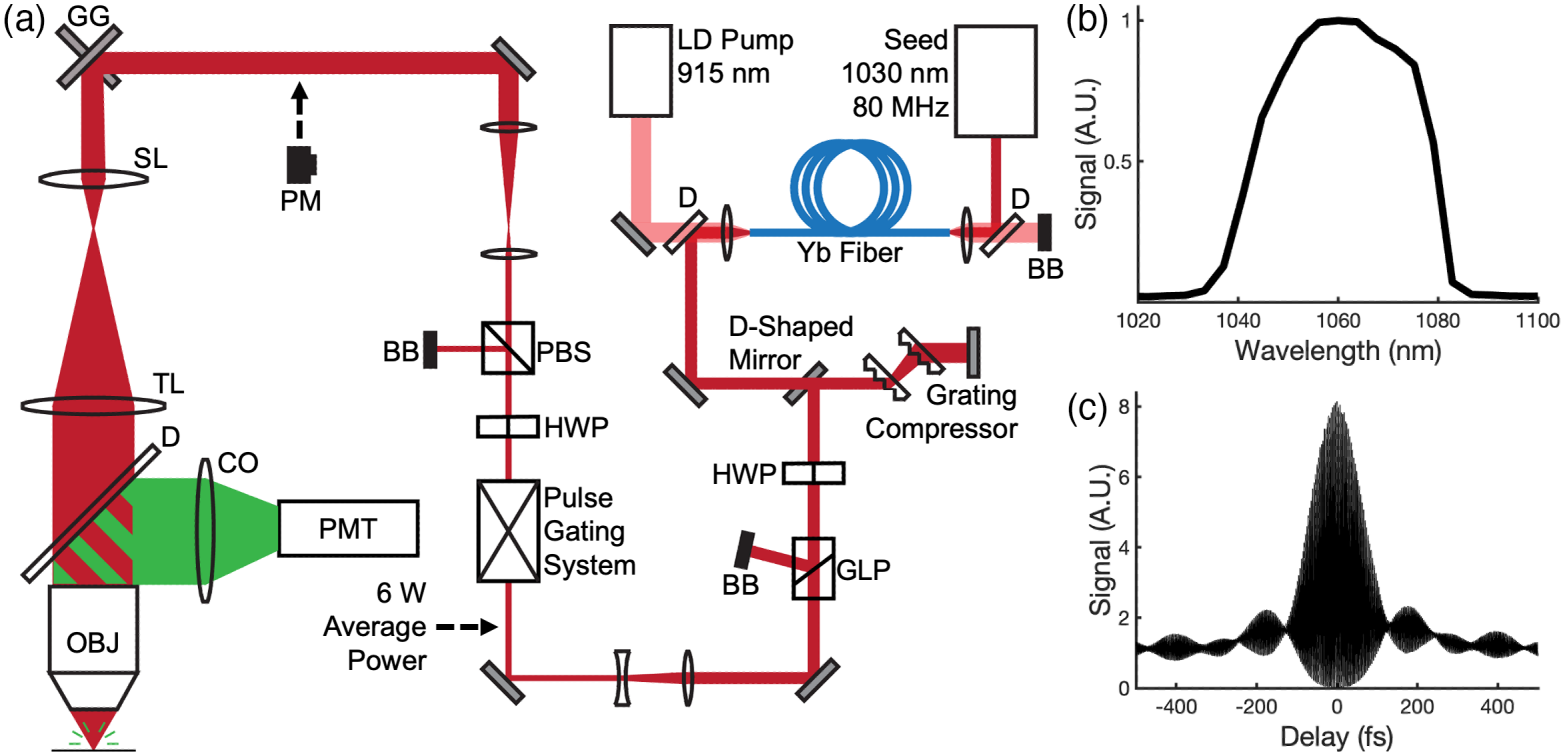
Schematic and characterization of the imaging system. (a) Schematic of the fiber amplifier, pulse gating system, and two-photon microscope. (b) Fiber amplifier spectrum. (c) *In situ* fiber amplifier autocorrelation measured through the objective. The pulse width is ∼110  fs assuming a ${\mathrm{sech}}^{2}$ shape. GG, galvo-galvo scanners; SL, scan lens; TL, tube lens; D, dichroic; OBJ, objective; CO, collection optics; PMT, photomultiplier tube; PM, power meter; BB, beam block; PBS, polarizing beam splitter; HWP, half-waveplate; GLP, Glan-laser polarizer; and LD, laser diode.

The complete excitation and microscopy setup is shown in Fig. 1(a). A custom ytterbium fiber amplifier served as the excitation laser for this work.^19^^,^^20^ The amplifier was constructed using 6 m of double-clad polarization-maintaining ytterbium-doped fiber. It is seeded by an 80 MHz commercial oscillator with a 100 mW average power (NKT Photonics, Origami 10-80) and pumped by a 30 W, 915 nm laser diode. Amplifier settings were selected to produce a beam with an average power of 6 W after pulse compression and dispersion compensation, which was accomplished using a pair of transmission gratings. Pulses are centered around λ=1060  nm [Fig. 1(b)], and *in situ* autocorrelation indicates a pulse width of 110 fs in the imaging plane that assumes a ${\mathrm{sech}}^{2}$ pulse shape [Fig. 1(c)]. Complete details of the Yb amplifier can be found in previous publications.^19^^,^^20^

**Fig. 2:**
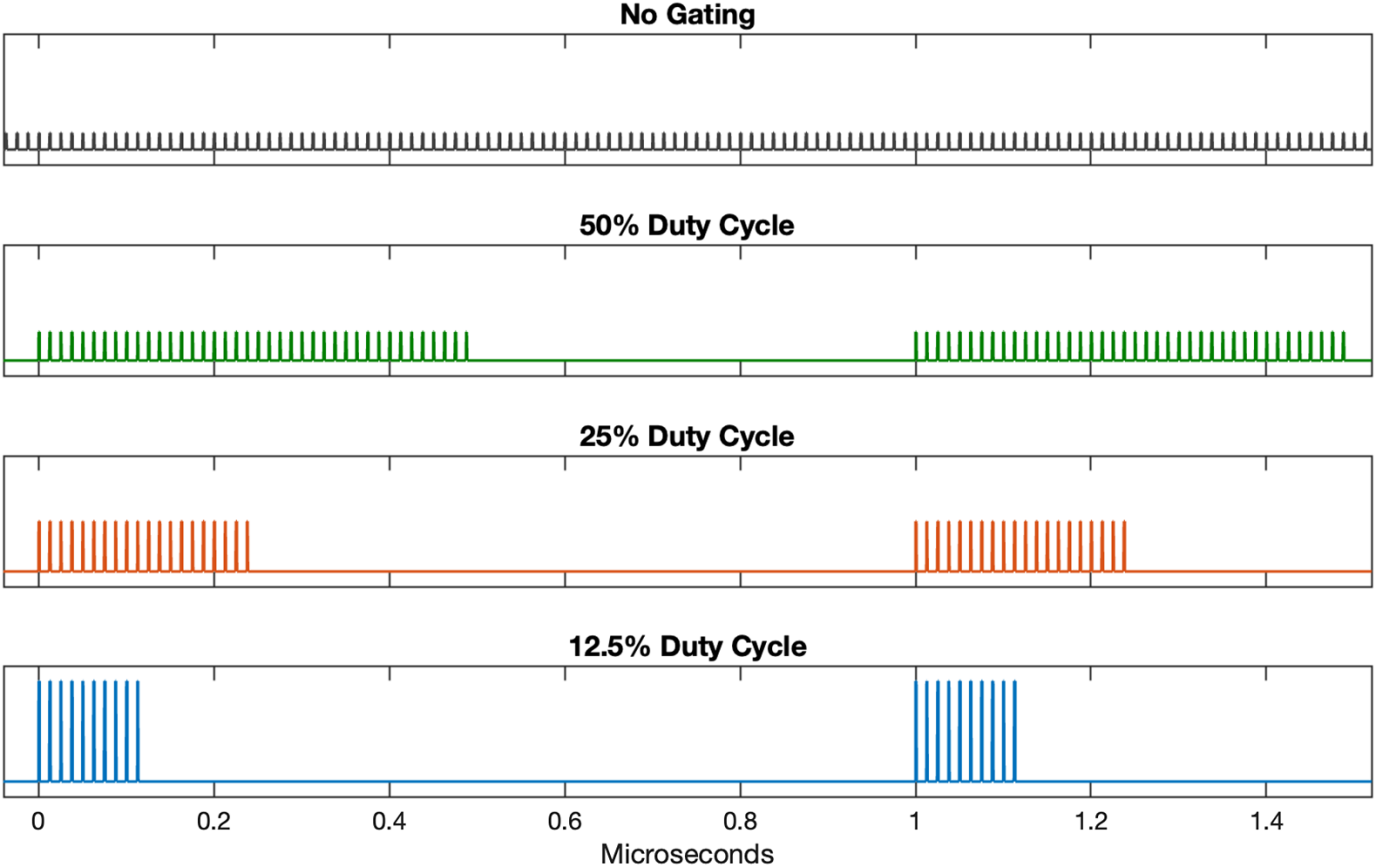
Ideal pulse trains used for imaging, all approximately with the same average power. There is a “no gating” condition in which the EOM transmitted all pulses in the 80 MHz fiber amplifier pulse train, as well as 50%, 25%, and 12.5% duty cycle conditions in which the EOM transmitted pulse trains in gates at a 1 MHz repetition rate.

Following compression, the beam is telescoped down to a diameter of ∼1.5  mm and sent through an electro-optic modulator (EOM) (Conoptics, 360-80) for pulse gating. The extinction ratio for rejected pulses is ∼100:1, and the throughput is ∼85% for transmitted light. The EOM is controlled by a driver (Conoptics, Model 25D), which either passes or rejects light based on a signal from a digital delay generator (Stanford Research Systems, DG645) triggered off the amplifier seed. The EOM control pulses were designed to pass gates of excitation pulses at a 1 MHz repetition rate, as shown in Fig. 2. The gate duration was set to achieve a duty cycle of either 50%, 25%, or 12.5%. A 25% duty cycle, for example, would cyclically pass 20 pulses and then reject 60 from the 80 MHz fiber amplifier pulse train. This has a similar effect to reducing the original 80 MHz repetition rate to 20 MHz, which was not possible given the timing limitations of the gating equipment used.

### 2.2 Multiphoton Microscope

A half-waveplate and polarizing beam splitter set the excitation power sent to the microscope following pulse gating. The beam was expanded to fill the microscope objective back aperture, which was either a 25× objective (Olympus XLPLN25XSVMP2, 1.0 NA) for *in vivo* imaging or a 10× objective (Nikon CFIPlan10×, 0.25 NA) for cuvette experimentation. The excitation beam was scanned using a pair of galvanometer mirrors (Thorlabs, QS7XY-AG) conjugated to the objective back aperture using a scan lens (Thorlabs, SL50-2P2, f=50  mm) and tube lens (Thorlabs, two AC508-400-C lenses in Plössl configuration, f=200  mm) combination. The backscattered fluorescent signal was directed to a photomultiplier tube (PMT) (Hamamatsu, H10770PB-50) with a 775 nm cutoff dichroic filter (Semrock, FF775-Di01) and was further filtered with a 609/181 bandpass filter (Semrock, FF01-609/181-25), which immediately preceded the PMT photocathode. Imaging was controlled using a custom LabVIEW software. Although it may be necessary to synchronize EOM gating with the data acquisition sampling in some situations, this was not done for this work given our low sampling rate (160 kHz) relative to the gating frequency (1 MHz).

### 2.3 Animal Protocols

All animal work was approved of by the University of Texas at Austin Institutional Animal Care and Use Committee. Adult female C57BL/6 mice were fit with cranial windows in a similar manner to that described in a previous publication.^21^ Carprofen (10  mg/kg, subcutaneous) and dexamethasone sodium phosphate (2  mg/kg, subcutaneous) were administered to control inflammation during the procedure, and mice were allowed to heal for at least 2 weeks prior to imaging. Temperature was maintained using a heating pad during both surgeries and imaging, and mice were anesthetized with isoflurane (2.5% induction and 1.5% maintenance). In all *in vivo* sessions, vasculature was labeled through 100  μL retro-orbital injections of 5% w/v 70 kDa dextran-conjugated Texas Red (Invitrogen, D1830) diluted in physiological saline. The excitation power was limited to 100 mW measured at the brain surface to avoid thermal damage.

## 3 Results

### 3.1 Signal Generation in a Cuvette as a Function of Excitation Duty Cycle

**Fig. 3:**
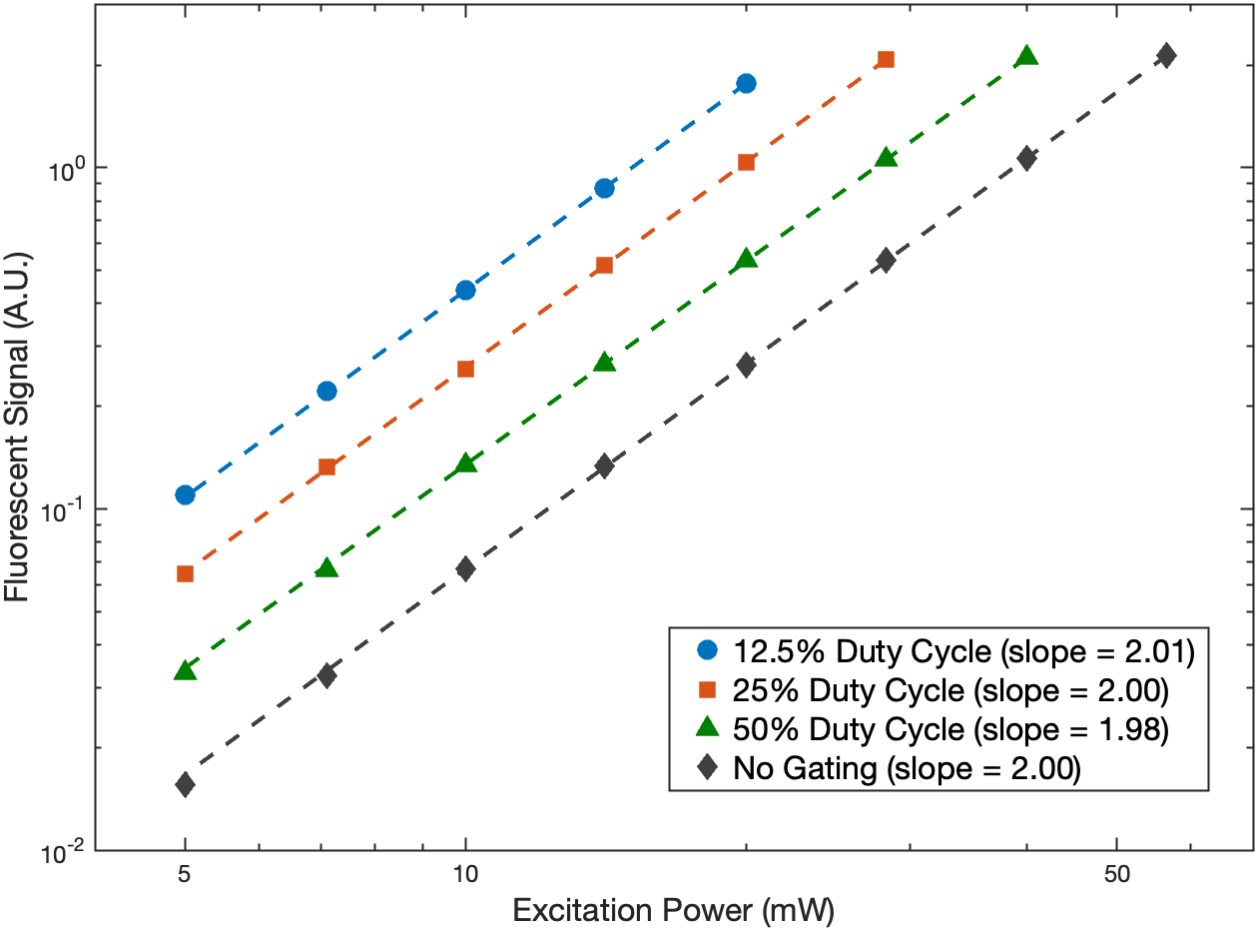
Fluorescent signal generated with different excitation duty cycles. Power was varied from 5 to a maximum of 56.6 mW measured before the microscope optics at the position shown in Fig. 1(a). The resulting fluorescence measurements are plotted with the pre-microscope power on a log-log scale. Linear fits were performed for each duty cycle (dashed lines), and the resulting slopes are included in the plot legend.

The pulse gating system was set to either transmit the complete 80 MHz pulse train without gating or to transmit pulse trains with a 12.5%, 25%, or 50% duty cycle at a 1 MHz repetition rate. The fluorescent signal generated within a cuvette containing a 39  μM Texas Red solution was measured for all gating conditions. The average excitation power for each state was 5 mW at a minimum [measured before the microscope optics at the position shown in Fig. 1(a)] and increased by factors of √2 until saturation was observed. Plots of the signal level measured for each excitation condition are shown in Fig. 3. When plotting fluorescence versus the excitation power on a log-log scale, linear fits reveal a slope of two for all conditions as expected for two-photon excitation. For the 50%, 25%, and 12.5% duty cycles, we see a 2.02, 3.97, and 6.73-fold average increase in signal respectively relative to the no gating condition. Figure S1 in the Supplementary Material shows how fluorescence varies when altering the duty cycle while maintaining the excitation pulse energy.

### 3.2 *In Vivo* Signal Generation Comparison Between Duty Cycles

For *in vivo* imaging, the pulse gating system was set to transmit pulse trains with either a 12.5%, 25%, or 50% duty cycle at a 1 MHz repetition rate. The 80 MHz ungated pulse train was not used here as the system required an elongated period for stabilization following a switch to this condition due to thermal effects within the EOM crystal (Fig. S2 in the Supplementary Material). First, a 12.5% duty cycle pulse train was used to image a three-slice stack (3  μm axial step size, eight frames averaged) with the power level set to avoid saturation. Stacks were then reacquired with this same duty cycle using 75% and 50% of the original power. This was followed by the acquisition of two final stacks both at the original power using a 25% and then a 50% duty cycle. This process was performed twice at approximate depths of 250 and 500  μm.

**Fig. 4:**
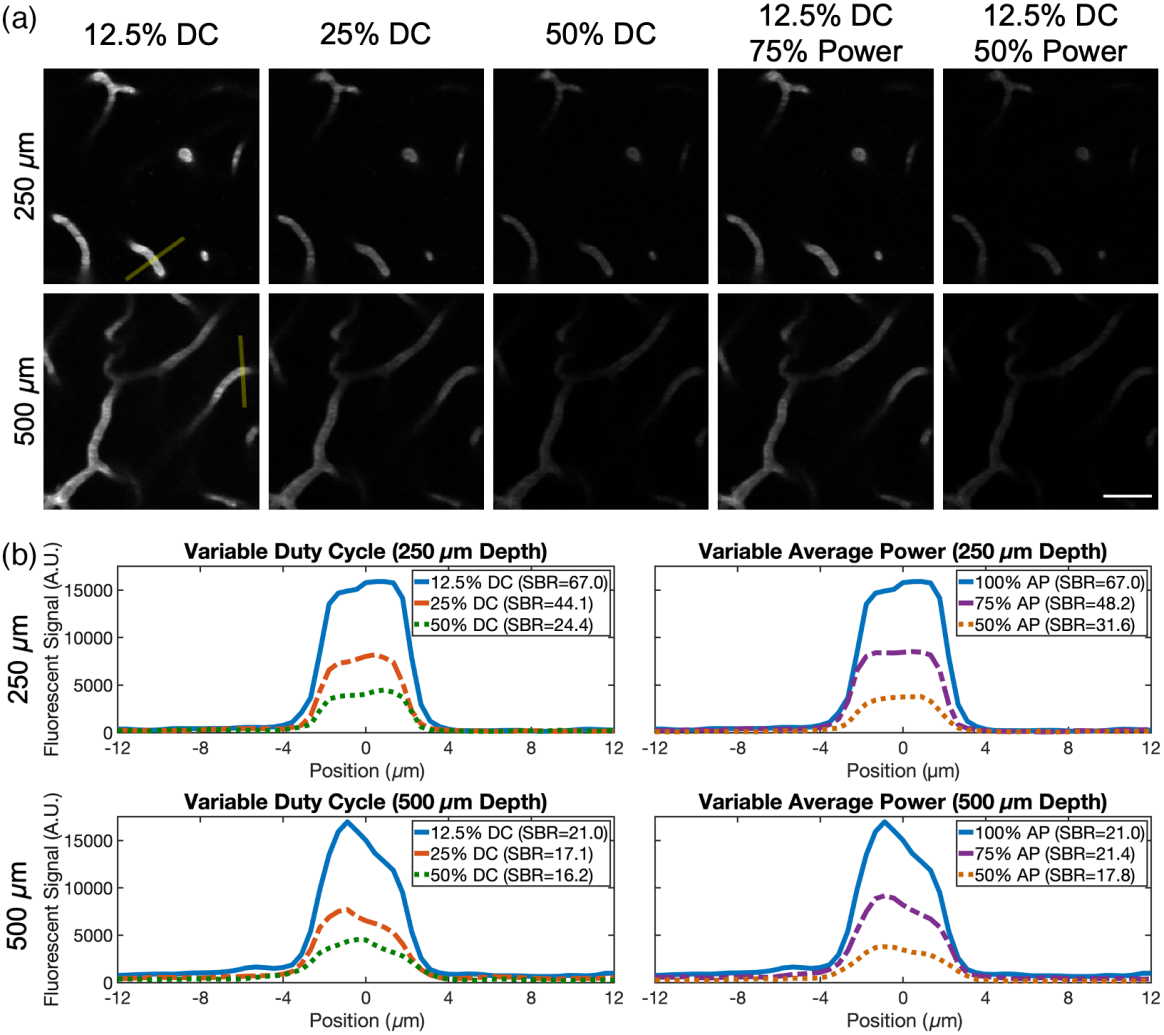
*In vivo* comparison of pulse delivery conditions. (a) Images of Texas Red-labeled vasculature acquired using varying excitation powers and duty cycles at approximately 250  μm (top) and 500  μm (bottom) deep. Each one is a three-image maximum intensity projection (3  μm axial step size). 5-pixel-thick lines for the vessels indicated in panel (a) were used to create the profiles in panel (b) and to calculate the SBR. The average power at the brain surface was held constant for the leftmost line profiles (7 mW for 250  μm, 31 mW for 500  μm), and the duty cycle was held constant for the rightmost line profiles (12.5% for 250  μm and 500  μm) in panel (b). Scale bar is 25  μm. DC, duty cycle and AP, average power.

Following acquisition, 1-pixel-radius median filters were applied to each image, and maximum intensity projections were created to ultimately use for analysis [Fig. 4(a)]. 5-pixel-thick, 80-pixel-long line profiles were created for the same three vessels in each projection at each depth. Figure 4(b) shows example profiles for the vessels indicated in Fig. 4(a). Profiles are shown for the additional vessels in Figs. S3 and S4 in the Supplementary Material. The SBR was determined with the profiles by dividing the peak value by the background, which was the average of the first 15 and last 15 pixels. Relative to the 25% and 50% duty cycles, the 12.5% duty cycle condition resulted in peaks that were on average 2.02±0.15 and 3.59±0.37 times greater in magnitude, respectively (± indicating standard error). Relative to the 75% and 50% average power conditions, the full power 12.5% duty cycle condition resulted in peaks that were on average 1.84±0.15 and 4.42±0.23 greater in magnitude, respectively. No major differences in relative peak signal generation between the excitation conditions are observed when comparing these two depths.

### 3.3 Pulse Gating for Deep Imaging

**Fig. 5:**
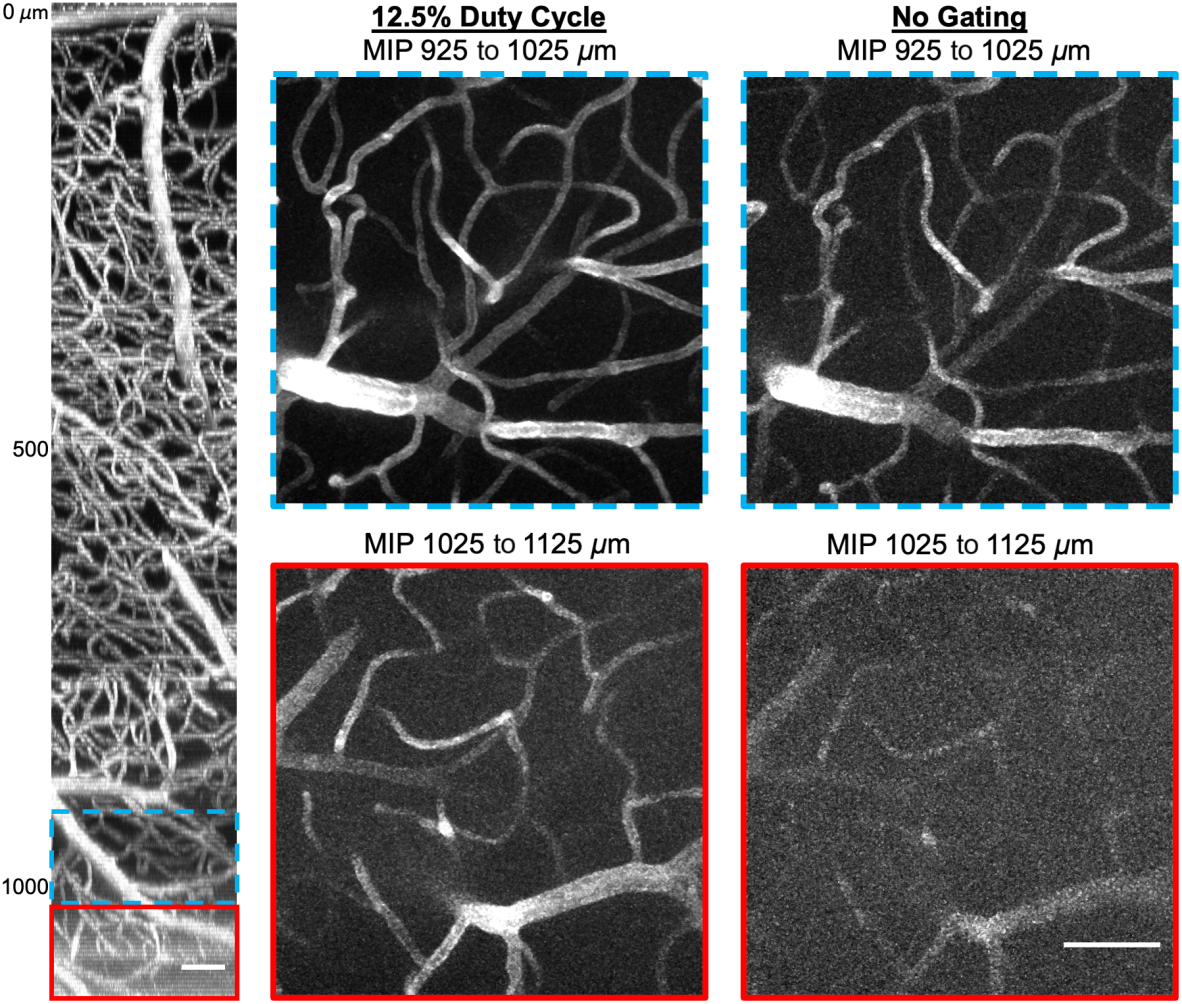
Deep imaging of Texas Red-labeled vasculature at a 12.5% duty cycle (middle) and without pulse gating (right). These maximum intensity projections (MIP) were recorded using a 100 mW average power measured at the brain surface. The side-projection (left) was imaged using <5  mW at the surface and up to 100 mW in deep regions. Scale bars are 50  μm. Scroll-throughs for maximum intensity projections are included in Video 1 (Video 1, MP4, 3.08 MB [URL: https://doi.org/10.1117/1.NPh.10.4.045006.s1]).

To investigate pulse gating when imaging deep structure, the 12.5% duty cycle pulse train was directly compared to the no gating condition. The EOM was first set to transmit the complete 80 MHz pulse train, and Texas Red-labeled neurovasculature was imaged in 5  μm axial step sizes until vessels were no longer clearly observed. Both frame averaging and average excitation power were increased with depth until 925  μm was reached. Then from 925 to 1125  μm, 10 frames were averaged together for each acquired image, and excitation power at the brain surface was maintained at 100 mW. The pulse gating system was then altered to operate at a 12.5% duty cycle, and EOM throughput was allowed to equalize for ∼10  min as a thermal equilibrium was approached (Fig. S2 in the Supplementary Material). Despite the thermal differences, pulse width was confirmed to be the same for each gating condition. Vasculature was then reimaged from 925 to 1125  μm using the same average power and frame averaging conditions as with the ungated pulse train. Maximum intensity projections through the resulting deep vascular stacks are displayed in Fig. 5, and scroll-throughs of the two stacks are included in Video 1.

**Fig. 6:**
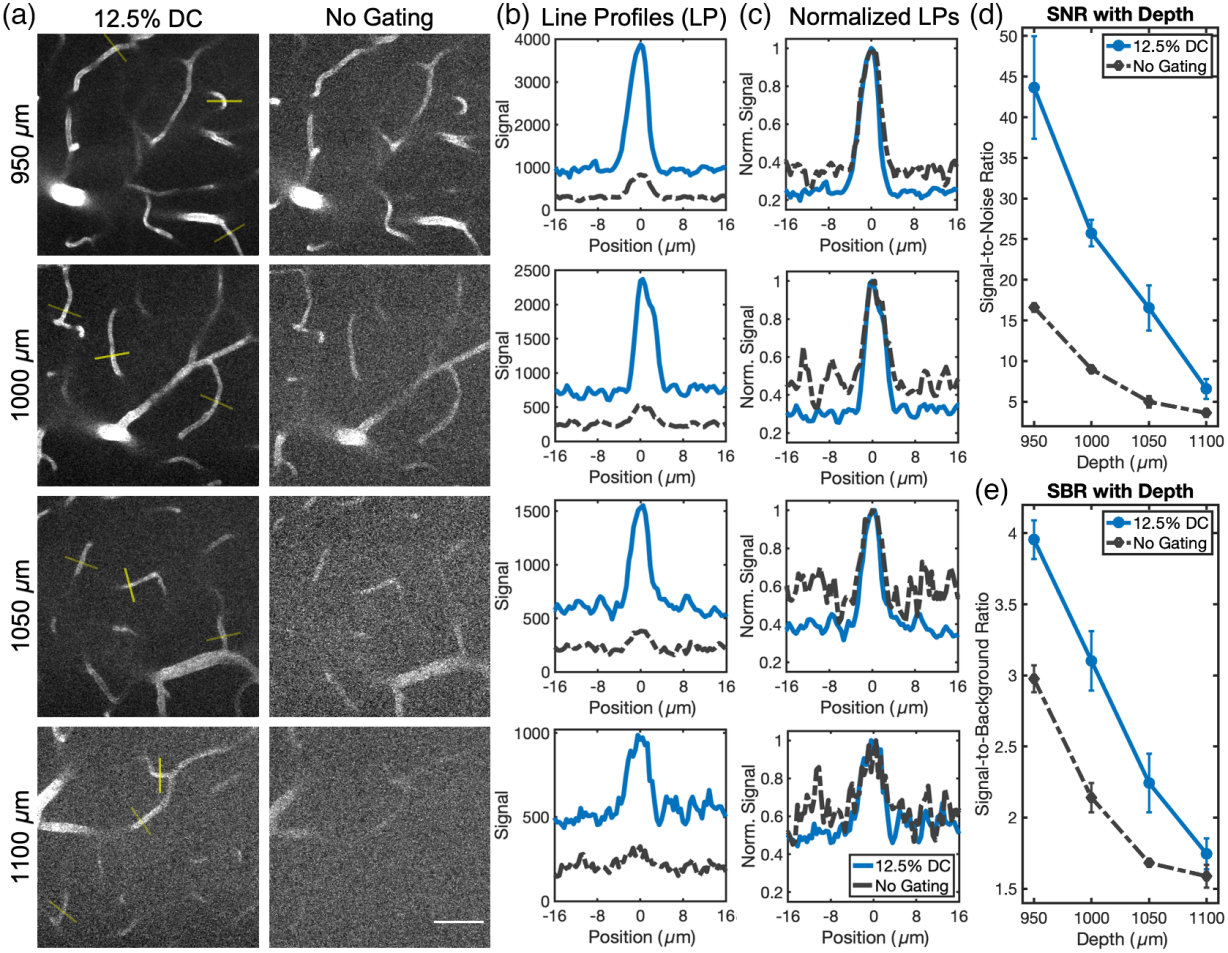
Deep imaging SNR and SBR comparisons between the 12.5% duty cycle and the ungated conditions. (a) Images used for quantifying the SNR and SBR. Each one is a three-image maximum intensity projection centered around the indicated depth (5  μm axial step size). (b) Line profiles through the vessels with the brightest markers shown in panel (a). Each plot corresponds with the depth listed in the same row. (c) Line profiles in panel (b) after normalization. (d) Average SNR with depth as determined from all vessels indicated in panel (a). (e) Average SBR with depth for all vessels marked in panel (a). Images are from the same stacks displayed in Fig. 5. Power at the brain surface was 100 mW for all images. Scale bar is 50  μm. Error bars represent standard error.

To quantify benefits of using a reduced pulse delivery, three representative vessels at 950, 1000, 1050, and 1100  μm were used to calculate both the SNR and SBR [Fig. 6(a)]. Similar to Sec. 3.2, the images for analysis were projections created using the image at the indicated depth along with those at the preceding and following depths. The SBR was calculated in the same manner previously described, and the SNR was calculated using the formula $(\mu_{\text{peak}}-\mu_{\mathrm{bkgr}})/\sigma_{\mathrm{bkgr}}$, where the 5 greatest values were averaged for $\mu_{\text{peak}}$ and the 15 first and 15 last pixels were grouped together and used for determining both $\mu_{\mathrm{bkgr}}$ and $\sigma_{\mathrm{bkgr}}$. A greater SNR and SBR are observed for all depths for the 12.5% duty cycle pulse train, as shown in Figs. 6(d) and 6(e). Table 1 lists the relative increase in these two metrics at each depth. The profiles for one vessel at each depth are shown in Fig. 6(b). Profiles for these same vessels are shown again in Fig. 6(c), following normalization. The normalized profiles for all remaining vessels are shown in Fig. S5 in the Supplementary Material. For both the SBR and SNR quantifications, $\mu_{\text{peak}}$ is the variable which changes most with depth.

**Table 1:** Relative SNR and SBR using a 12.5% duty cycle pulse gating strategy compared with imaging with no gating. Values were calculated using the line profiles of the three vessels indicated in Fig. 6 at each depth. Values after the ± indicate standard error.

	950 μm depth	1000 μm depth	1050 μm depth	1100 μm depth
12.5% duty cycle SNR/no gating SNR	2.64 ± 0.40	2.87 ± 0.20	3.34 ± 0.48	1.83 ± 0.11
12.5% duty cycle SBR/no gating SBR	1.33 ± 0.08	1.45 ± 0.06	1.34 ± 0.13	1.09 ± 0.01

## 4 Discussion

To verify proper system behavior, it is helpful to calculate the theoretically expected enhancements in the signal magnitude with pulse gating. Fluorescence intensity (F) for two-photon excitation is proportional to the square of pulse energy, $F\propto E^{2}$. When reducing the 80 MHz pulse train using a 12.5% duty cycle, we would expect an eight-fold increase in signal when imaging with the same average power, as shown by comparing

$$F_{0}\propto E_{0}^{2},$$ (1)

$$F_{12.5\%}\propto \frac{1}{8}{\left(8E_{0}\right)}^{2}=8E_{0}^{2},$$ (2)

where $F_{0}$ and $E_{0}$ are the fluorescence intensity and pulse energy for the ungated pulse train, respectively, and $F_{12.5\%}$ is fluorescence intensity for the 12.5% duty cycle gating. Here we assume that the pulse energy is increased by 8, but the number of pulses is reduced by a factor of 8 because the average power remains constant. Equation (2) assumes that the pulse characteristics are constant between the two different conditions. Equation (2), however, does not account for the imperfect extinction and transmission of the EOM. To get a more accurate pulse energy relationship for our system (1:100 extinction ratio, 85% transmission), the following must be set equal:

$$P_{0}\propto E_{0},$$ (3)

$$P_{12.5\%}\propto \frac{1}{8}E_{12.5\%}+\frac{7}{8}(\frac{1}{100\times 0.85})E_{12.5\%}=\frac{1}{8}E_{12.5\%}+\frac{7}{8}(\frac{1}{85})E_{12.5\%},$$ (4)

where $P_{0}$ and $P_{12.5\%}$ are average powers. This yields a relationship of $E_{12.5\%}\approx 7.39\;E_{0}$. Taking this, we modify Eq. (2) as

$$F_{12.5\%}\propto \frac{1}{8}(7.39E_{0}{)}^{2}+\frac{7}{8}{\left(\frac{7.39}{85}\times E_{0}\right)}^{2}\approx 6.83E_{0}^{2},$$ (5)

where we now expect a 6.83-fold increase in signal magnitude for the 12.5% duty cycle compared to the ungated condition [Eq. (1)]. Experimentally, we found a similar increase of 6.73 when exciting Texas Red in solution, verifying that our pulse gating strategy improves the signal generation as expected.

Theoretical predictions then suggest that the 12.5% duty cycle offers a 3.50-fold increase in signal generation relative to the 50% duty cycle and a 1.83-fold increase compared with the 25% duty cycle. We found similar 3.33-fold and 1.69-fold increases respectively during cuvette testing and 3.59-fold and 2.02-fold increases in peak signal during superficial vascular imaging. We made use of this increase to demonstrate two useful applications of pulse gating for *in vivo* imaging. First, reducing the duty cycle allows for imaging with lower average powers to limit tissue heating while maintaining high signal levels (demonstrated in Sec. 3.2). Second, images can be acquired with a reduced pulse delivery at the same average power to better resolve features. We use this strategy to improve deep vascular imaging in Sec. 3.3, where we observe consistent increases in both the SBR and SNR. This can help researchers accurately identify vessels in images to better vectorize neurovascular networks.^22^^,^^23^ Note that the SBR will approach 1 at the same depth for all gating conditions. This suggests that the relative benefit of pulse reduction will decrease with depth, which seems to be confirmed in Table 1, where the smallest improvement in the SBR is seen at the deepest location analyzed (1100  μm). That said, many vessels that are nearly indistinguishable here when imaging with the ungated 80 MHz pulse train are resolved when switching over to the 12.5% duty cycle excitation pattern. This can in some part be attributed to the improved SNR offered alongside the raised SBR.

One limitation to consider is how systems like the one presented here may limit the imaging speed. Excitation pulses must be delivered at a rate faster than the pixel dwell time to properly sample each region during two-photon microscopy. Our system as currently constructed may not be compatible with fast imaging tools such as resonant scanning mirrors due to the maximum control gate repetition rate. This compatibility can be improved by replacing our digital delay generator with timing electronics that produce control pulses at a faster rate. One such example is the Model 305 Synchronous Countdown System manufactured by Conoptics, which creates control pulses at up to a 30 MHz repetition rate when paired with the additional components in our system. We do not require such a timing system, however, as the two-photon microscope used in this work is driven by standard galvanometer scanners. Therefore, we can effectively image while using a relatively low pulse gate repetition rate (1 MHz).

This work should encourage other groups to adopt excitation systems similar to ours for deep imaging applications in which potential thermal damage is a concern. The gating strategy that we demonstrate maintains a high pulse energy that is necessary for deep imaging in scattering media while limiting the average power. These results are unique for the gating of high-power pulse trains that otherwise cannot be used for *in vivo* imaging without the risk of excessive tissue heating. Although similar strategies can be used with other standard 80 MHz two-photon microscopy sources, such as Ti:sapphire oscillators, with average powers of 1 to 2 W, these sources do not often operate at powers where damage is likely when imaging deep tissue structure. Consequently, pulse energy is also relatively low for pulse trains from these sources compared with that provided by our fiber amplifier. The ability to image with pulse gates at such a low duty cycle (12.5%) while maintaining the needed average and peak powers for deep two-photon microscopy is therefore ultimately enabled by the relatively high average power (∼6  W) of our custom ytterbium amplifier. Also of importance, the amplifier was built in our lab and can be replicated relatively easily, and the entire excitation system is relatively cost-efficient^19^^,^^20^ compared with commercial alternatives that provide similar pulse energies. The imaging strategy can be modified to further limit the average power by synchronizing the pulse delivery to only occur while scanning discrete regions of interest in a similar manner to Li et al.^24^ Although the excitation system used by Li et al. has an advantage over ours in that it can excite fluorophores through either three-photon or two-photon excitation, ours may be simpler to implement and is therefore more accessible. Our two-photon excitation wavelength is also slightly longer (1060 nm compared with 920 nm), which further assists with deep imaging when paired with appropriate fluorophores.

## 5 Conclusion

In this work, we used an EOM-based pulse gating system to produce up to a 6.73-fold increase in two-photon signal generation while maintaining the excitation power. We additionally demonstrated that pulse gating improved *in vivo* neurovascular two-photon imaging by raising both the SBR and SNR. We demonstrated an average 1.37-fold increase in the SBR and a 2.95-fold increase in the SNR when imaging from 950 to 1050  μm in depth with 12.5% duty cycle pulse train gating as opposed to using a constant 80 MHz pulse train with the same average power. At around a 1100  μm depth, the pulse gating helps resolve vessels that are otherwise indistinguishable for the frame averaging conditions used. The results presented here should encourage the use of pulse gating as a viable method to either mitigate thermal damage through reducing average excitation power or to improve the SNR and SBR through allowing heightened excitation pulse energies.

## Code, Data, and Materials Availability

Raw images and data are not publicly available at this time but may be obtained from the authors upon reasonable request.
