# Supplementary material for: Pulse train gating to improve signal generation for in vivo two-photon fluorescence microscopy
Source: Neurophotonics. 2023 Nov 6;10(4):045006. doi: 10.1117/1.NPh.10.4.045006 (PMC10627479; doi:10.1117/1.NPh.10.4.045006)
Supplement: Supplementary file 1 [file NPh_010_045006_SD001.pdf]

Supplemental Information - Pulse train gating to improve signal generation for *in vivo* two-photon fluorescence microscopy

Figure S1

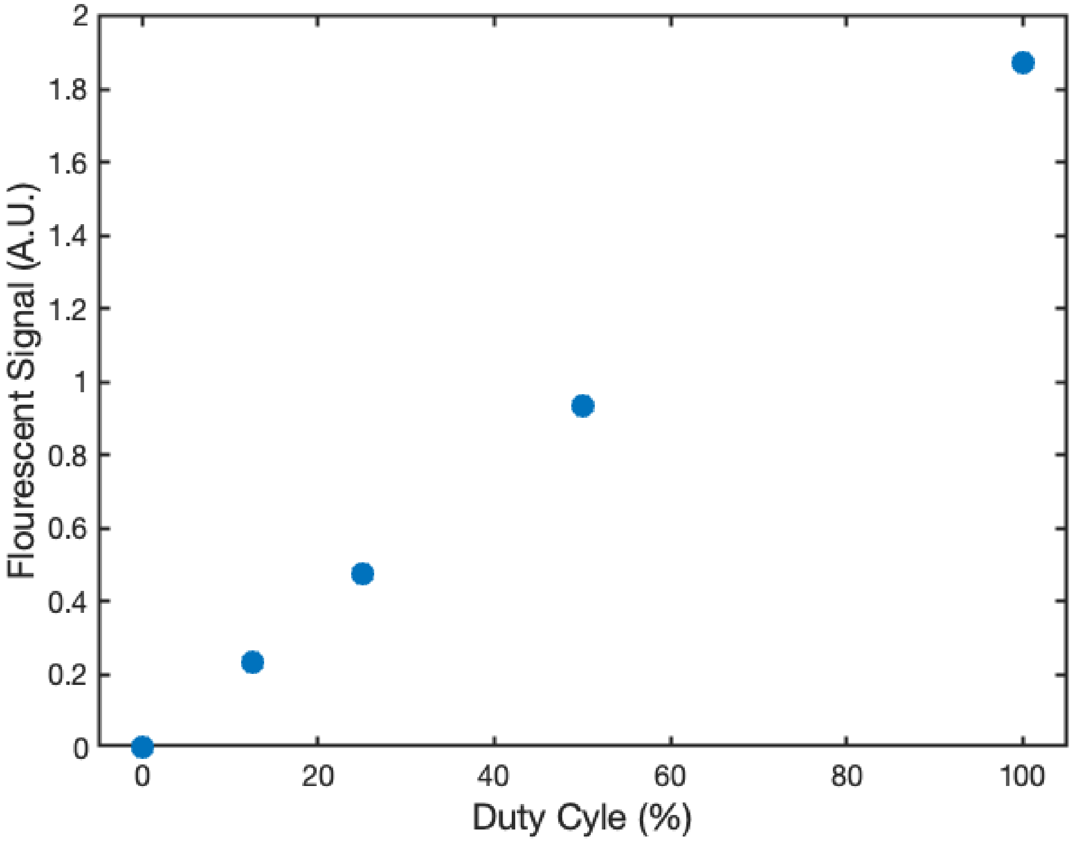

Fig. S1 Fluorescent signal generated in a cuvette as duty cycle is adjusted. Power at the EOM input was held constant and power in the imaging plane was adjusted only through altering the duty cycle.

Figure S2

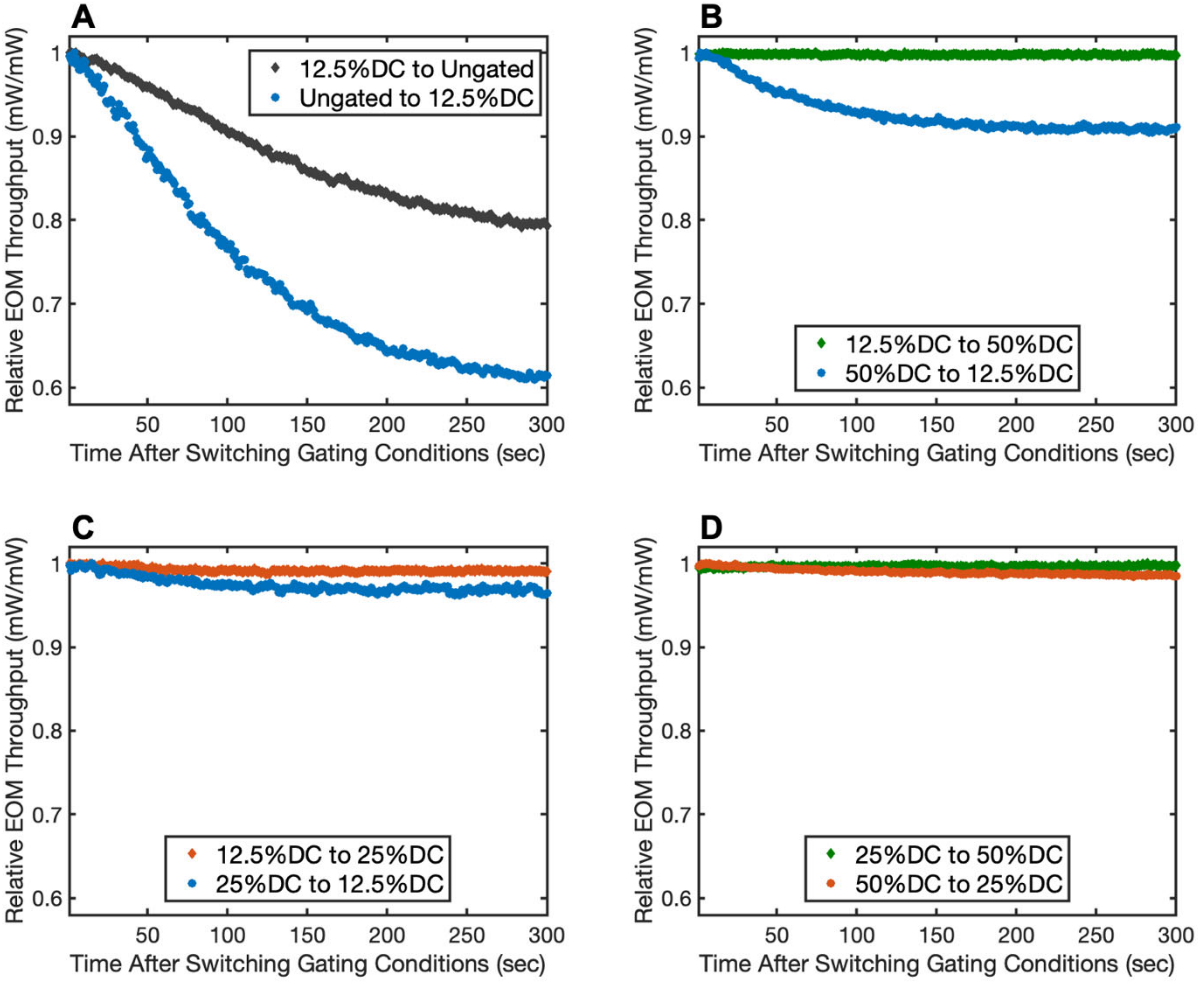

Fig. S2 EOM throughput with time when switching gating conditions. Each curve is independently normalized to its initial power measurement. (A) Switching from a 12.5% duty cycle to an ungated condition and vice versa. (B) Switching from a 12.5% duty cycle to a 50% duty cycle and vice versa. (C) Switching from a 12.5% duty cycle to a 25% duty cycle and vice versa. (D) Switching from a 25% duty cycle to a 50% duty cycle and vice versa.

Figures S3 and S4

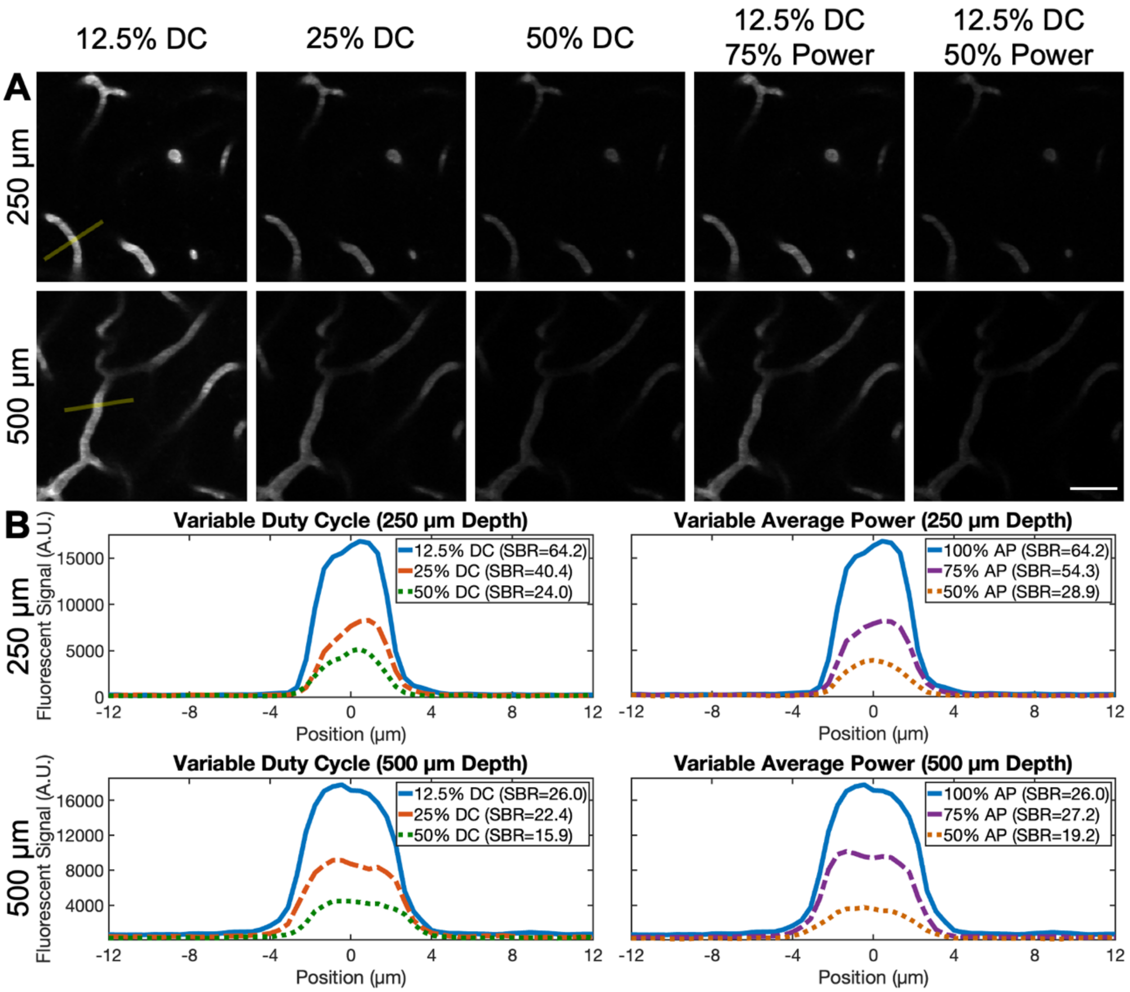

Fig. S3 Second group of vessels (A) and line profiles (B) for Sec. 3.2.

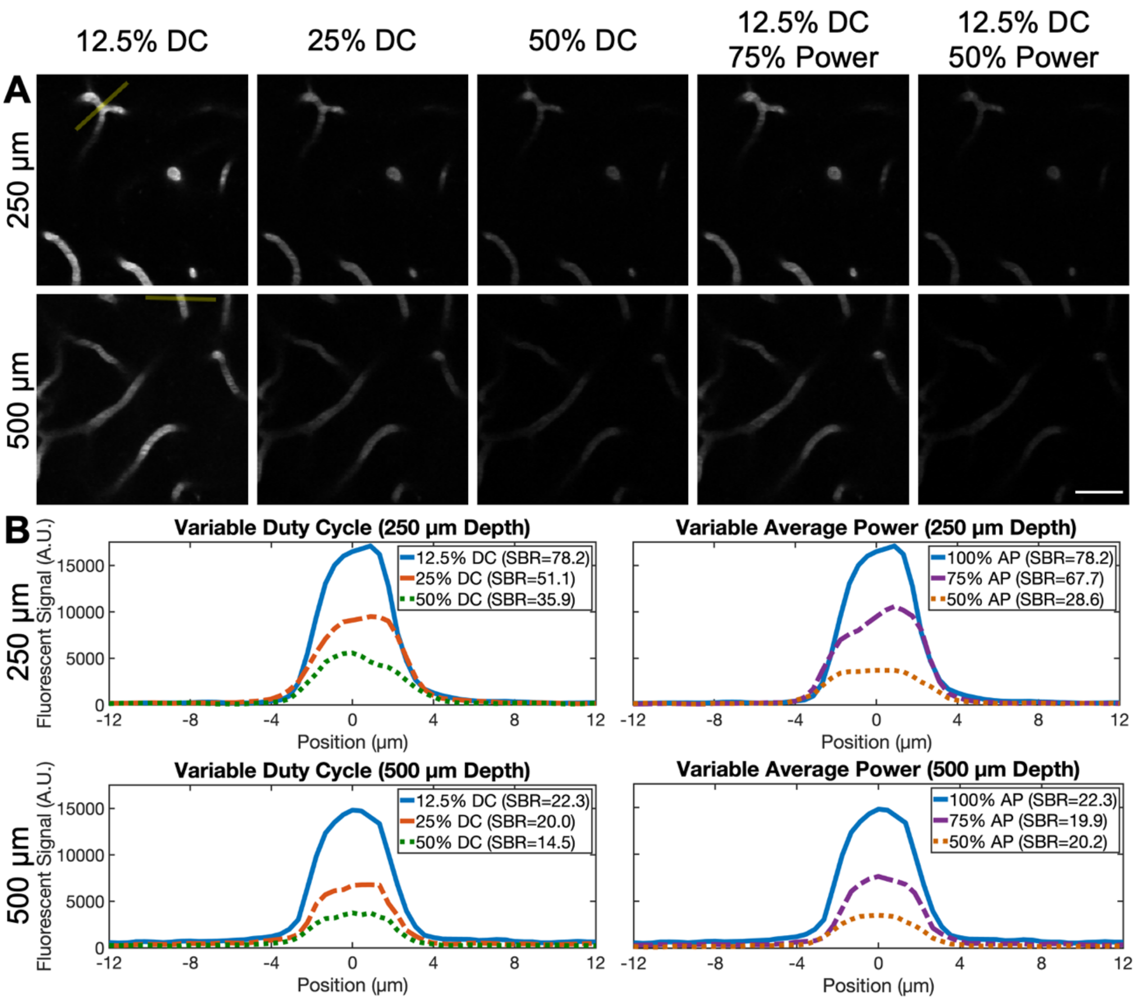

Fig. S4 Third group of vessels (A) and line profiles (B) for Sec. 3.2

.

Figure S5

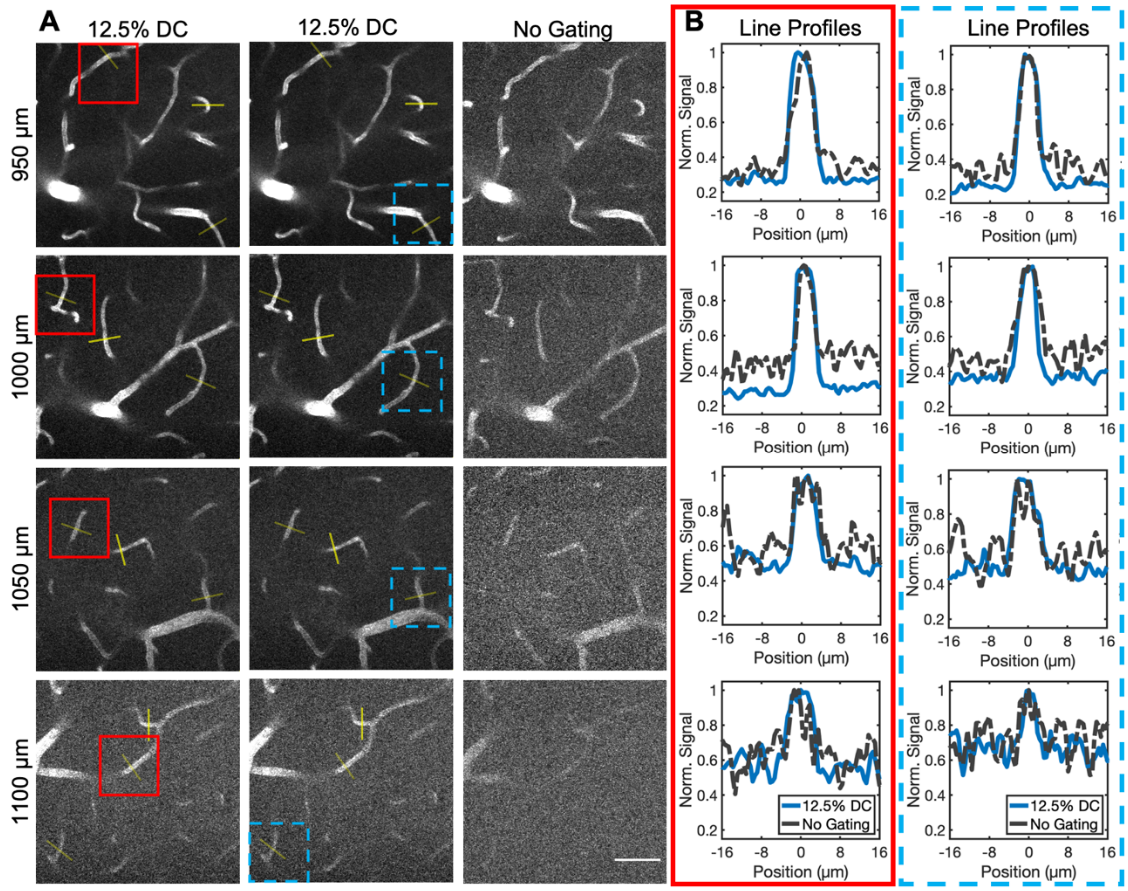

Fig. S5 Images (A) and additional normalized line profiles (B) that go with Sec. 3.3.
